# Supplementary material for: Nicastrin-Like, a Novel Transmembrane Protein from Trypanosoma cruzi Associated to the Flagellar Pocket
Source: Microorganisms. 2021 Aug 17;9(8):1750. doi: 10.3390/microorganisms9081750 (PMC8400621; doi:10.3390/microorganisms9081750)
Supplement: Supplementary file 1 [file microorganisms-09-01750-s001.zip › microorganisms-1266568-supplementary.pdf]

Supplementary Figure S1:

F1	MLRRRMVCMGGCPR
F2	MVCMGGCPRGPTEW
F3	GCPRGPTEWTSIFV
F4	PTEWTSIFVLWIAA
F5	SIFVLWIAAWLMTC
F6	WIAAWLMTCGATAE
F7	LMTCGATAERGFSG
F8	ATAERGFSGDIPPA
F9	GFSGDIPPAPHDAA
F10	IPPAPHDAAYAAYF
F11	HDAAYAAYFRNYPI
F12	AAYFRNYPIVRPCI
F13	NYPIVRPCIMKAVF
F14	RPCIMKAVFMAVKS
F15	KAVFMAVKSDDQSN
F16	AVKSDDQSNNSVAY
F17	DQSNNSVAYRGCSI
F18	SVAYRGCSIADNAV
F19	GCSIADNAVAPHGL
F20	DNAVAPHGLLLLHA
F21	PHGLLLLHATEVAQ
F22	LLHATEVAQASMDG
F23	EVAQASMDGCEDNA
F24	SMDGCEDNAPSLQD
G1	EDNAPSLQDIIRGL
G2	SLQDIIRGLSIPDT
G3	IRGLSIPDTVFSSG
G4	IPDTVFSSGIGLVL
G5	FSSGIGLVLSTRDK
G6	GLVLSTRDKDDYEE
G7	TRDKDDYEEGGREN
G8	DYEEGGRENRVKTN
G9	GRENRVKTNTQYDM
G10	VKTNTQYDMTCFLA
G11	QYDMTCFLAAVQHY
G12	CFLAAVQHYNAVAN
G13	VQHYNAVANKHTQG
G14	AVANKHTQGLMPPI
G15	HTQGLMPPITAVAF
G16	MPPITAVAFSDDNR
G17	AVAFSDDNRCCRFS
G18	DDNRCCRFSGDRRS
G19	CRFSGDRRSGEELL
G20	DRRSGEELLLPREV
G21	EELLLPREVRSVYD
G22	PREVRSVYDFVILY
G23	SVYDFVILYFPSAA
G24	VILYFPSAATSSSG

H1	PSAATSSSGPANSD
H2	SSSGPANSDWTALM
H3	ANSDWTALMNAAGF
H4	TALMNAAGFNRIHW
H5	AAGFNRIHWAEGRR
H6	RIHWAEGRRYPQNV
H7	EGRRYPQNVLETRN
H8	PQNVLETRNGMSLA
H9	ETRNGMSLAELLSS
H10	MSLAELLSSPTRVR
H11	LLSSPTRVRSCLEG
H12	TRVRSCLEGPSPSC
H13	CLEGPSPSCVPLSG
H14	SPSCVPLSGWTVWT
H15	PLSGWTVWTSTADM
H16	TVWTSTADMRWEWN
H17	TADMRWEWNETSLN
H18	WEWNETSLNFEKKT
H19	TSLNFEKKTRKGAV
H20	EKKTRKGAVALLVA
H21	KGAVALLVASTAVS
H22	LLVASTAVSLVQDA
H23	TAVSLVQDATPGAD
H24	VQDATPGADCPASA
I1	PGADCPASAIVATL
I2	PASAIVATLSVLEA
I3	VATLSVLEALRRVG
I4	VLEALRRVGGDDSR
I5	RRVGGDDSRDVYAF
I6	DDSRDVYAFFFPGE
I7	VYAFFFPGEHVGSV
I8	FPGEHVGSVGSARF
I9	VGSVGSARFISDAT
I10	SARFISDATMLECV
I11	SDATMLECVHAGLS
I12	LECVHAGLSNCTAL
I13	AGLSNCTALAYKEK
I14	CTALAYKEKLNFTT
I15	YKEKLNFTTVDFNA
I16	NFTTVDFNAIDTFL
I17	DFNAIDTFLVVDQV
I18	DTFLVVDQVAYQDA
I19	VDQVAYQDAPLYYH
I20	YQDAPLYYHVDSRV
I21	LYYHVDSRVGKTGS
I22	DSRVGKTGSAQQQK
I23	KTGSAQQQKAELEM
I24	QQQKAELEMEKSGV

J1	ELEMEKSGVRRAST
J2	KSGVRRASTTRLPY
J3	RASTTRLPYSPITT
J4	RLPYSPITTLLDLL
J5	PITTLLDLLPAVMA
J6	LDLLPAVMAGEKVF
J7	AVMAGEKVFLTLTR
J8	EKVFLTLTRYNTTF
J9	TLTRYNTTFANPDV
J10	NTTFANPDVFTVVD
J11	NPDVFTVVDKTAEH
J12	TVVDKTAEHSALRP
J13	TAEHSALRPASVAE
J14	ALRPASVAEAADVM
J15	SVAEAADVMLRVLL
J16	ADVMLRVLLPPTQN
J17	RVLLPPTQNAAETP
J18	PTQNAAETPPVTSV
J19	AETPPVTSVNRSLV
J20	VTSVNRSLVEQLWG
J21	RSLVEQLWGCFTEN
J22	QLWGCFTENLQCKF
J23	FTENLQCKFLSAPN
J24	QCKFLSAPNDVAEF
K1	SAPNDVAEFMAPDY
K2	VAEFMAPDYSVGEM
K3	APDYSVGEMANSRI
K4	VGEMANSRITDTQA
K5	NSRITDTQAAIEAA
K6	DTQAAIEAALHRIG
K7	IEAALHRIGWTDVA
K8	HRIGWTDVARSPAV
K9	TDVARSPAVPKSLR
K10	SPAVPKSLRIPHGD
K11	KSLRIPHGDWGATW
K12	PHGDWGATWEQDRD
K13	GATWEQDRDWMRLH
K14	QDRDWMRLHNDSRY
K15	MRLHNDSRYELHVM
K16	DSRYELHVMSFWRG
K17	LHVMSFWRGNIGAR
K18	FWRGNIGARSTMVG
K19	IGARSTMVGSDTVA
K20	TMVGSDTVALIFLM
K21	DTVALIFLMLSITA
K22	IFLMLSITATISLT
K23	SITATISLTLCIHV
K24	TISLTLCIHVCVRK

Supplementary Figure S2

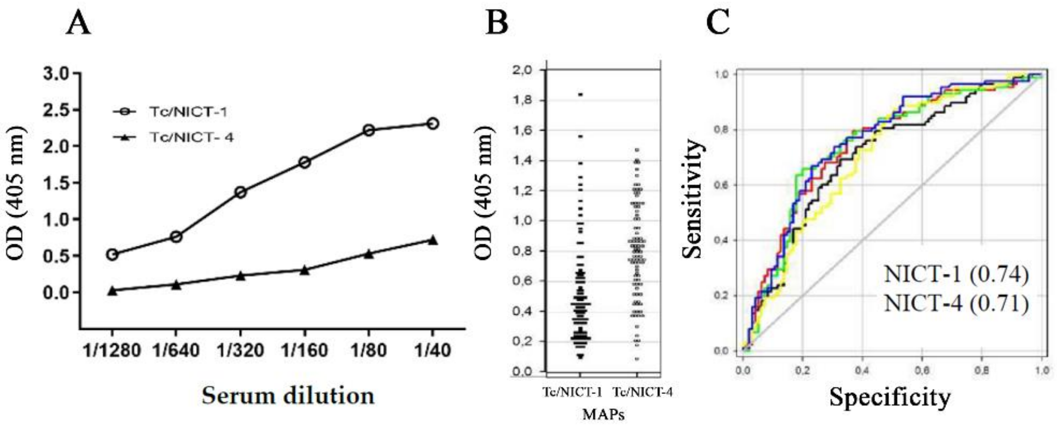

Supplementary Figure S3:

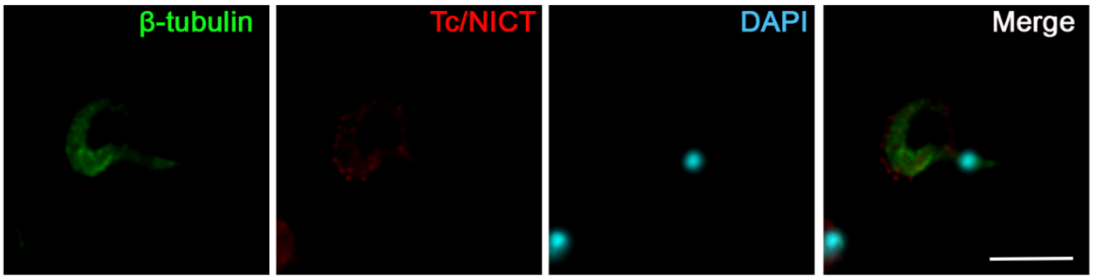

Supplementary Figure S4:

A B

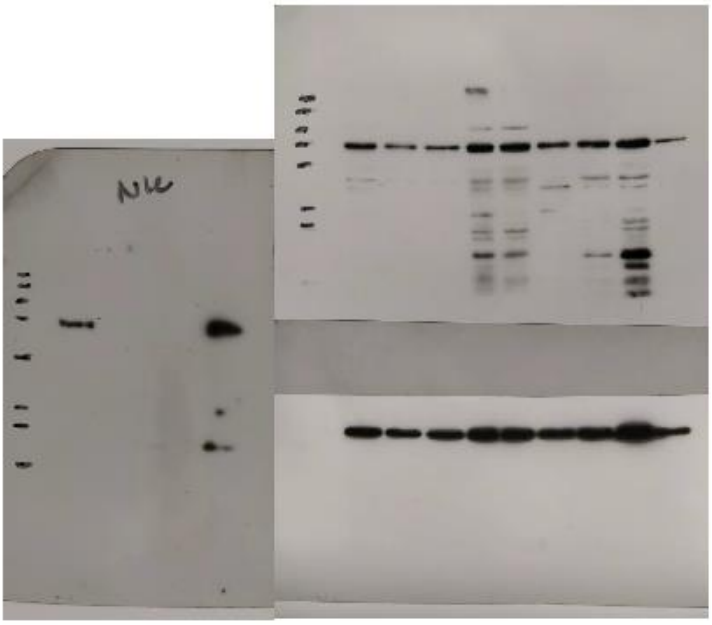

C

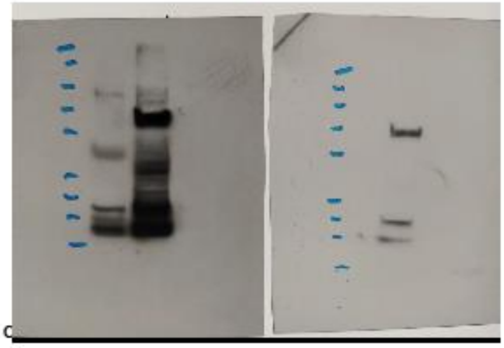

Supplementary Figure S5:

MLRRRMVCMGGCPRGPTEWTSIFVLWIAAWLMTCGATAERGFSGDIPPAPHDAAYAAYFRNYPIVRPCIM
KAVFMAVKSDDQSNNSVAYRGCSIADNAVAPHGLLLLHATEVAQASMDGCEDNAPSLQDIIRGLSIPDTV
FSSGIGLVLSTRDKDDYEEGGRENRVKTNTQYDMTCFLAAVQHYNAVANKHTQGLMPPITAVAFSDDNRC
CRFSGDRRSGEELLLPREVRSVYDFVILYFPSAATSSSGPANSDWTALMNAAGFNRIHWAEGRRYPQNVL
ETRNGMSLAELLSSPTRVRSCLEGPSPSCVPLSGWTVWTSTADMRWEWNETSLNFEKKTRKGAVALLVAS
TAVSLVQDATPGADCPASAIVATLSVLEALRRVGGDDSRDVYAFFFPGEHVGSVGSARFISDATMLECVH
AGLSNCTALAYKEKLNFTTVDFNAIDTFLVVDQVAYQDAPLYYHVDSRVGKTGSAQQQKAELEMEKSGVR
RASTTRLPYSPITTLLDLLPAVMAGEKVFLTLTRYNTTFANPDVFTVVDKTAEHSALRPASVAEAADVML
RVLLPPTQNAAETPPVTSVNRSLVEQLWGCFTENLQCKFLSAPNDVAEFMAPDYSVGEMANSRITDTQAA
IEAALHRIGWTDVARSPAVPKSLRIPHGDWGATWEQDRDWMRLHNDSRYELHVMSFWRGNIGARTMVGSD
TVALIFLMLSITATISLTLCIHVCVRK

Position	Residue	Score
84	NNS	1.095587
329	NET	0.982870
425	NCT	0.838883
436	NFT	1.117064
526	NTT	1.157668
580	NRS	0.724098
675	NDS	0.815644
